# Cocaine in Surgery

**Published:** 1888-12

**Authors:** Freidrich Haenel


ARTICLE III.

### COCAINE IN SURGERY.

BY DR. FREIDRICH HAENEL.

[Abstract of a Lecture Delivered in the Society for Natural and Medical Science of Dresden, April 14, 1888.]

Cocaine is sufficiently appreciated and applied, not only by specialists, but also by practical physicians, on account of its local anæsthetizing action on the conjunctiva and the cornea of the eye and on the mucous membranes of the nose, of the oral cavity, of larynx, then of the male and female urogenital apparatus. But this is not the case in the same degree with regard to the application of the remedy to the anæsthetization of the epidermis. Yet, it is just to the practical physician who is often in a position to perform small surgical operations without competent assistance, cocaine offers such inestimable advantages that it may not seem superfluous to point out parenchymatous injections of cocaine solution for the anæsthetization of circumscribed portions of the epidermis and of deeper layers of tissue.

The idea of using this medicament, which had proved an excellent anæsthetic for mucous membranes, in the same way for local anæsthetization of the epidermis, was not a very remote one, and, indeed, almost simultaneously, experiments in this respect have been instituted and published from different sides.

In Germany it was *Landerer*, who first, in Centralblatt fur Chirurgie, 1885, No. 48, called attention to local anæsthetization by means of subcutaneous injections of cocaine and related the successful results obtained. He used a 4 per cent. solution, injecting from two to three lines of a Pravas syringe, equal to 0.008=0.12 cocaine, and after an interval of five minutes he was able to perform small operations, without the patients giving expression to any pains. Some time later, *Wolfler's* paper was published, in which *Landerer's* favorable results were confirmed, the only difference being that Wolfler had employed somewhat larger doses, as high as 0.05 cocaine. A short time before this, the method had been successfully employed in North America, and from there, from Corning, the advice was given, in all cases where it was feasible, to combine it with artificial vacuity of blood, causing in this way a longer duration of cocaine action. In France, *Verchere* had used already the subcutaneous application of cocaine, and *Rusconi* in Milan had obtained anæsthesis, sufficient for painless performance of incisions, by daubing the untouched skin with cocaine solution.

These first essays were soon followed by numerous others, which, although differing considerably in their statements about concentration and quantity of the fluid to be injected, were unanimous on the excellence and suitableness of the method.

After a previous failure, I have applied it, during the last six months, in about 80–100 cases (half of which concerned extraction of teeth), mostly in polyclinic practice, and with very satisfactory result.

The proceeding is a very simple one, after thorough disinfection of the whole field of operation, the epidermis; in the direction of the incision to be made, is subjected to one or more injections, inserting the canule of the common Pravas syringe obliquely into the skin, not introducing it under the skin, and then pushing it ahead a little space, so as to control the largest possible field with one single insertion. It is desired to anæsthetize deeper portions, the same place of insertion allows the addition of an injection into the subcutaneous tissue, or even deeper. I mostly use a 5 per cent solution, which is more reliable than weaker solutions; yet even with 3 per cent. solutions, I have had favorable results. On account of the easy decomposition of the solutions and their liability to mould, it is to be recommended never to keep on hand large quantities of the solution and always to add a few drops of carbolic acid. I have endeavored to succeed with the smallest possible doses, and the largest quantity of cocaine, which I ever used, was 0.025, or one-half syringe of a 5 per cent. solution. This was a case in which the operation, under vacuity of blood, lasted one hour. Ordinarily, I did not exceed from two to three lines of the syringe for one injection.

The cocainized field, which is characterized by immediate anæmia and in a short time spreads out somewhat, has the size of a Pfenning to a Mark, according to the quantity of the liquid used. Anæsthesis sets in rapidly and is complete after five to ten minutes, continues, when connected with artificial vacuity of blood for an hour and more, with free circulation of blood for 15 to 25 minutes or even less, always according to the quantity of the solution injected.

The cases in which cocaine anæsthesis was applied, were: incisions of furuncles; phlegmonic abscesses, fissures of fistular channels, extirpations of small tumors, extraction and excision of extraneous bodies, amputations and exarticulations of fingers and toes, operations of grown-in nails, extraction of teeth.

Application of cocaine mostly prevented pain completely, sometimes there was some pain, but of a decidedly less degree, and in a very few instances only, it would entirely give way. In these cases, however, there was mostly some technical defect to be noticed. Thus, in opening a phlegmatic abscess through the very thin epidermis, the injection had manifestly been pushed too far down into the abscess itself.

For extraction of teeth, I use cocaine in the following way: I practice an injection of 0.005 gr. cocaine on each side of the tooth between gum and alveola, and then, after about 5 minutes, I apply the forceps. If the patient at this moment still experiences some pain, I wait for a while. In a great number of cases, the effect was complete, *i. e.*, the patients did not experience any pain at all. In other cases of about the same number, a partial result only was to be noticed. Application and raising of the forceps was not felt in a painful manner, while the extraction itself was painful, although in a mitigated degree. In a small number of cases, in about six, cocaine had no result at all.

There are a great many very favorable reports on other operations performed under cocaine anæsthesis, even larger operations, such as operations of phimosis, division of fistula of the rectum, herniotomy, tarcheotomy in adults, extirpation of tuberculous lymphatic glands of the neck, uranoplastic and staphylorrhaphic, radical operation of hydrocele, laparatomy, gastrotomy, mammillary amputations of the upper and of the lower part of the thigh, etc.

However, for operations on the bone, cocaine is not sufficient. Patients who supported incisions in fleshy parts without manifestation of pain, will, according to my experiences, feel strong pains in manipulations of the periosteum and of the bones.

For this reason and then on account of the necessity of employing, in these extensive operations, two large doses of cocaine, which in many cases have given rise to alarming toxicological phenomena, cocaine is not suitable for larger operations, or at least very exceptionally only, in cases for instance, where chloroform should be counter-indicated. Nor is the psychical element to be overlooked. The patient, who sees and hears the proceedings of the operation, becomes easily excited, especially in a serious and protracted operation, and in such cases humanity alone will cause chloroform to be preferred. Besides this, the motility of the patient will often interfere, and in operations, requiring a relaxation of the muscular system, total narcosis will always be preferable. For the above reasons it is obvious that cocaine anæsthesis is not applicable in surgical operations of children, or at least in a very limited measure.

Concerning the dangerous feature of cocaine, slight cases of intoxication—I only refer to acute intoxication, chronic intoxication not coming into account in this mode of application of cocaine—are not rare, and even observations on serious, alarming phenomena are extant, as these reported by Mayerhausen, Bock, Schilling, Heymann, Mannheim, Robson, Kilham, Comanos Bey. A prominent instance is the well-known case of death caused by injection into the rectum of 1.2 cocaine, having driven the physician, Kolomnin in St. Petersburg, to commit suicide. Another fatal case is said to have occured in Warsaw in dental practice.

Slighter intoxications, the symptoms consisting in pallor, cold sweat, vertigo, heaviness and fatigue in the legs, sensation of anxiety, increased frequency of pulse, have been observed by me twice after application of 0.008 and 0.01 cocaine. In one of these cases when nitrate of amyl was employed as an antidote, as frequently recommended, it rendered very good service in this quality.

I had an opportunity of observing a severe case of intoxication towards the end of February of the current year. A dentist, according to his statement, had injected under the gums of a 19-year-old girl of strong constitution, although slightly chlorotic, 0.11 cocaine, ¾ syringe of a 15 per cent. solution in two portions, which was followed by the painless extraction of the carious tooth. The patient then became pale, fell backward and the whole body was seized with convulsions of great violence.

These epileptiform convulsions continued for 5 hours with short interruptions, and during this time the patient was entirely unconscious, giving no sign of reaction to any excitation. The pupils were moderately wide and without reaction. The pulse, not to be counted in the beginning, subsequentiy showed a frequency of 176; the frequency of respiration was 44. After the convulsion had ceased, the patient lay quiet, unconscious. When awaking, she was unable to walk; she was only able to sit in a squatting position; she could not raise the arm; she had an intense aversion for light, diminished sensibility of the skin, the mucous membranes, the nose and the oral cavity, complete loss of smell and taste, dryness and burning in the throat, thirst; then appeared, at first less conspicuously, but increasing to excessive height in the next days, a condition of pronounced casdialgia; to this was to be added retention of urine during twenty-four hours, complete lack of appetite during four days. While the other phenomena disappeared after two to three days it took forty hours before she could walk with trembling knees—cardialgia continued for six days. There were no permanent consequences to be noted. Nitrite of amyl had no visible effect in this case.

Severe cases of intoxication of this kind will necessarily lead to an understanding on the maximum doses which should be allowed. *Landerer* has proposed 0.015 as maximum doses. *Decker* (Munchoner med. Wochenschrift 1887, No. 39) 0.02. Possibly, somewhat larger quantities, as high as 0 03, may be given without inconvenience, provided the limit of what is allowed, especially in consideration of the individually different sensibility for cocaine, is not reached in decrepid persons, in patients with severe affections of the heart, and in persons reduced by pains, loss of blood and exhausting suppuration.

Slight phenomena of intoxication, for which nitrate of amyl is an excellent antidote of prompt action, are scarcely to be avoided and may well be submitted to in consideration of the great advantages presented by cocainc, and of the long consequential phenomena often induced by chloroform narcosis, not to mention chloroform collapse and chloroform death.—*Korrespondeuzblatt d arztl K. u. B. Vereine im Konigreich Scichsen.*

---

ARTICLE IV.

